# Dynamic Changes in Lung Function and Imaging in Patients with COVID-19

**DOI:** 10.1155/2022/1728446

**Published:** 2022-03-11

**Authors:** Lingyan Ye, Bingyu Hu, Shuangxiang Lin, Meifang Chen, Yicheng Fang, Susu He

**Affiliations:** ^1^Department of Respiratory Medicine, Taizhou Hospital of Zhejiang Province Affiliated to Wenzhou Medical University, Linhai, Zhejiang, China; ^2^Department of Radiology, Taizhou Hospital of Zhejiang Province Affiliated to Wenzhou Medical University, Linhai, Zhejiang, China

## Abstract

**Purpose:**

To investigate the recovery of lung function and chest imaging in patients with COVID-19 three months after clinical cure and discharge and the correlation between them.

**Methods:**

This study collected 80 patients diagnosed with 2019-nCoV infection who were discharged from the Taizhou Public Health Medical Center in Zhejiang Province between January 31, 2020, and March 10, 2020. Lung function examinations and lung CT scans were performed at discharge and three months after discharge. The dynamic changes examined at discharge and three months after discharge were observed, and their correlation was analyzed. All data collection ended on June 25, 2020.

**Results:**

Among the 80 COVID-19 patients discharged from the hospital, the rate of abnormality indicated by lung CT images was 97.5%, mainly presenting as patchy shadows (95%), ground-glass shadows (75%), grid-like lesions, interlobular septal thickening or fiber strip shadows (30%), consolidation shadows, and nodules (10 cases each). At discharge, 72 patients (90%) had pulmonary dysfunction, 64 (80%) had restrictive ventilatory dysfunction, and 48 (60%) had small airway dysfunction. Three months after discharge, the rate of abnormality indicated by lung CT images was 12.5%. Eight cases (10%) showed residual patchy shadows, but the density was weak, and the scope was reduced. Two cases (2.5%) showed nodular shadows. Three months after discharge, 18 patients (22.5%) had residual restrictive ventilatory dysfunction, 28 patients (35%) had small airway dysfunction, and 32 patients (40%) had diffuse dysfunction. Moreover, patients with more severe chest imaging manifestations (bilateral lesions and ground-glass shadows combined with interstitial lesions) also had more obvious lung function impairment.

**Conclusion:**

Three months after being clinically cured, patients with COVID-19 had good chest imaging absorption and no residual fibrosis. Some patients had mild to moderate pulmonary dysfunction, mainly restricted ventilation dysfunction, small airway dysfunction, and diffuse dysfunction.

## 1. Introduction

In December 2019, a pneumonia of unknown cause occurred in Wuhan [1], which was named “COVID-19” by the World Health Organization (WHO). COVID-19 is caused by the severe acute respiratory syndrome coronavirus-2 (SARS-CoV-2) [2]. The disease is mainly transmitted through the respiratory tract, with a rapid onset, strong infectivity, and a high fatality rate. At present, a pandemic with high morbidity and mortality has emerged globally [3, 4], seriously threatening people's safety and health. The common symptoms of COVID-19 are fever, dry cough, and fatigue, and some patients develop shortness of breath and dyspnea one week after onset [5, 6]. Previous studies have reported [7] that infection with Middle East respiratory syndrome (MERS) and acute respiratory syndrome (SARS), both *β* coronaviruses, can cause pulmonary fibrosis. A follow-up of the lung function of surviving patients also found that some patients had residual lung function impairment, including ventilatory function and diffusion dysfunction. Residual lung lesions are significantly correlated with lung function impairment in patients [8].

At present, there are also reports [9] that patients with COVID-19 have impaired diffusion function and restricted ventilation when discharged from the hospital, but there is a lack of relevant research on the dynamic changes in lung function of patients with COVID-19 and its correlation with lung imaging. This study aims to clarify the changes in lung function and imaging in patients with novel coronavirus infection and to evaluate the correlation between imaging characteristics and lung function impairment, which is of great significance for further understanding the outcome of COVID-19 and guiding pulmonary rehabilitation treatment in the later stage of COVID-19 recovery.

### 1.1. Patient Selection and Research Methods

Patient selection: eighty patients with COVID-19 who were hospitalized at the Taizhou Public Health Center in the Zhejiang Province between January 31, 2020, and March 10, 2020, were included in this study. Inclusion criteria: ① all respiratory secretions obtained from nasopharyngeal swabs tested positive for novel coronavirus by real-time PCR before admission, in compliance with the diagnostic criteria of China's COVID-19 diagnosis and treatment protocol [10]. ② All patients receiving oxygen therapy and mechanical ventilation were discharged after receiving routine treatment. [10] That is, their temperature returned to normal for at least 3 days, their respiratory symptoms significantly improved, and the nucleic acid test for the novel coronavirus was negative for 2 consecutive tests (with an interval of more than 24 h). Exclusion criteria: ① individuals with pulmonary diseases such as tuberculosis, lung cancer, and bronchiectasis were excluded by pulmonary imaging examination; ② pregnant patients were also excluded. The diagnostic criteria for severe COVID-19 patients are as follows: (1) respiratory distress, RR > 30 beats/min; (2) average oxygen saturation ≤93%, arterial oxygen partial pressure/oxygen concentration (PaO_2_/FiO_2_) ≤ 300, and (3) progression of chest imaging lesions >50% within 24–48 hours [11].

Methods: lung function tests and lung CT scans were performed on the day of discharge and three months after discharge, and the changes in lung function and lung CT features were compared and analyzed. We retrospectively collected data on sex, age, smoking history, past disease history, and other data of all patients, all of which were collected from electronic medical records until June 25, 2020. This study was approved by the Ethics Committee of the Enze Hospital of the Zhejiang Enze Medical Group (Center), and the enrolled patients provided written informed consent after retrospective data collection.

### 1.2. Pulmonary Function Test

All patients underwent lung function tests in accordance with the 2017 American Thoracic Society (ATS) and European Respiratory Society (ERS) lung function guidelines [12]. The PowerCube Body BF pulmonary function tester manufactured by GANSHORN Medizin Electronic (Germany) was used, and all pulmonary function tests were performed by the same technician. Lung function detection indicators included inspiratory vital capacity (IVC), forced vital capacity (FVC), forced expiratory volume in the first second (FEV1), ratio of forced expiratory volume in the first second to inspiratory vital capacity (FEV1/IVC), maximal expiratory flow rate at 25% of vital capacity (MEFR 25), maximal expiratory flow rate at 50% of vital capacity (MEFR 50), maximal expiratory flow rate at 75% of vital capacity (MEFR 75), and diffusion capacity for carbon monoxide (DLCO). All patients were required to take three tests to obtain the most consistent standard waveform. Except for FEV1/FVC, other lung function indices were expressed as the percentage of measured values to predicted values. Lung function assessment criteria included the following: FVC% or IVC% <80% is restricted ventilation dysfunction, FEV1/FVC <70% is obstructive ventilation dysfunction, MEFR 25–75% <70% is small airway dysfunction, and DLCO% <80% is diffuse dysfunction [13, 14].

### 1.3. Chest CT Protocols

All lung CT examinations were performed on the same day as the pulmonary function examinations. All images were obtained on one of the three CT systems (CT 780, United Imaging, China; Optima 660, GE, America; Somatom Definition AS+, Siemens Healthineers, Germany) with patients in the supine position. The main scanning parameters were as follows: tube voltage = 120 kV, automatic tube current modulation = 100–200 mAs, and slice thickness = 1.25–5 mm. All images were then reconstructed with the same incremental slice thickness in millimeters. Two chest radiographers with more than 5 years of experience described the main CT features (ground-glass, patchy, consolidation, grid-like, interlobular septal thickening, and nodular) and lesion distribution (left, right, or bilateral lungs).

### 1.4. Statistical Analysis

Categorical variables are expressed as frequencies and percentages, while continuous variables are presented as the mean ± standard deviation and median. *P* < 0.05 was considered statistically significant. All statistical analyses were performed using SPSS version 22.0 software.

## 2. Results

### 2.1. Patient Characteristics

Among the 80 patients with COVID-19, there were 42 males and 38 females, with an average age of 45.86 ± 11.23 years; 56 cases (70%) were the common type, 24 cases (30%) were the severe type, and 4 cases had a smoking history. There were 2 cases that had a history of chronic obstructive pulmonary disease, 14 cases (17.5%) that had at least one potential comorbidity, 4 cases (5%) that suffered from hypertension, 10 cases (12.5%) that had diabetes mellitus, and all patients had no history of cardiovascular and cerebrovascular diseases or malignant tumors. Except for 6 asymptomatic patients, the other patients presented with fever, cough, expectoration, chest tightness, or fatigue. The average duration of symptoms was 7.21 ± 2.06 days. All patients underwent lung CT scanning at admission, received confirmation of the existence of pneumonia, and achieved clinical cure after routine treatment. There were no complications, such as respiratory failure or impairment of other organ functions. The length of hospital stay was 23.93 ± 7.16 days (Table 1).

### 2.2. Pulmonary Function

Among 80 COVID-19 patients, 72 (90%) had pulmonary dysfunction at discharge: 66 (82.5%) had ventilation dysfunction, 2 (2.5%) had obstructive ventilation dysfunction, and 64 (80%) had restrictive ventilation dysfunction. Forty-eight patients (60%) presented with small airway dysfunction, of whom 42 patients (52.5%) had restricted ventilation dysfunction combined with small airway dysfunction. Three months after discharge, 28 patients (35%) had pulmonary dysfunction, and all of them had small airway dysfunction, among which 18 patients (22.5%) had restricted ventilation dysfunction combined with small airway dysfunction. DLCO was measured in 80 patients with COVID-19 after three months of cure and discharge; 32 patients (40%) had diffuse dysfunction, and 24 patients (30%) had complications with small airway dysfunction (Table 2). The results in Table 2 show that the abnormal pulmonary function of patients with COVID-19 is mainly characterized by restrictive ventilation dysfunction, small airway dysfunction, and diffusion dysfunction, and the pulmonary function impairment of severe patients is more common than that of ordinary patients. The pulmonary function indices IVC, FEV1, FVC, MEFR 25, MEFR 50, and MEFR 75 of 80 patients on the day of discharge were 62.6 ± 13.8, 83.7 ± 12.1, 84.3 ± 12.9, 72.3 ± 31.4, 81.9 ± 27.3, and 90.4 ± 19.2, respectively. These indices changed significantly three months after discharge, 77.8 ± 16.9, 90.4 ± 12.5, 92.1 ± 18.9, 78.3 ± 35.5, 90.5 ± 24.4, and 99.1 ± 22.8, respectively. There was a significant difference between the two (*P* < 0.05) (Table 3), indicating that the pulmonary function decline of COVID-19 patients gradually recovered compared with pulmonary function at discharge, and the pulmonary function of most patients returned to normal.

### 2.3. Lung CT Manifestations (Table 4)

Among the 80 COVID-19 patients, lung CT examination showed normal lung performance in 2 cases (2.5%) upon discharge, and residual lung lesions in the remaining 78 cases (97.5%) were as follows: there were 76 cases of patchy shadows (95%), 60 cases of diffuse or scattered ground-glass changes (75%), 20 cases of consolidation shadows (25%), 24 cases of interstitial lesions (grid-like lesion and interlobular septal thickening or fiber strip shadows), and 20 cases of nodular manifestation (25%). Sixty cases (75%) had lesions in both lungs. Lung CT examination three months after discharge showed that 70 cases (87.5%) showed normal lung appearance, with ground-glass shadows, mesh shadows, and interlobular septal thickening and other lesions having been completely absorbed; 8 cases (10%) showed residual patchy shadows, but the density became weak and the scope was reduced; and 2 cases (2.5%) showed nodularshadows.

### 2.4. Comparison of the Correlation between Lung CT and Lung Function (Table 5 and Figures 1 and 2)

**Figure 1:**
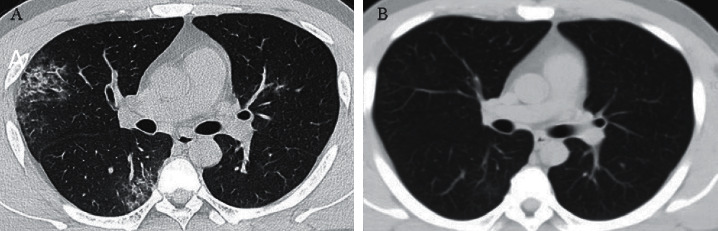
(a) At discharge, lung CT showed multiple patchy and flocculent high-density shadows in both lungs, with blurred edges, especially in the periphery. Some of them showed ground-glass changes and thickened interlobular septa. (b) The lung lesions were absorbed by reexamination three months after discharge.

**Figure 2:**
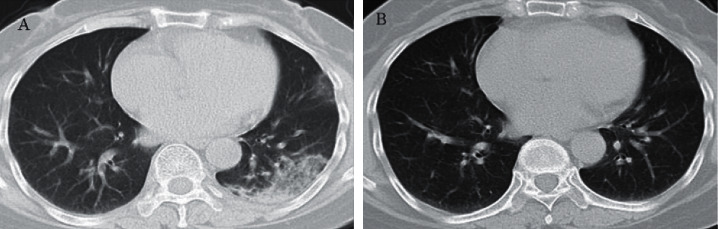
(a) At discharge, lung CT showed high-density and patchy shadows in the left lung with blurred edges. (b) The lung lesions were absorbed by reexamination three months after discharge.

There was no statistically significant difference in FEV1, FVC, or IVC between the two pulmonary lesions and the single pulmonary lesion, while there was a statistically significant difference between MEFR25 and MEFR50 (*P* < 0.05) (Table 5). Therefore, there was no statistically significant difference in pulmonary ventilation function between the single lung lesions and the double lung lesions. The degree of impairment of small airway function in double lung disease is more serious than that in single lung disease. At the same time, we found that the decline in FEV1, FEV1/FVC, IVC, MEFR25-75, and other indicators of lung function was more obvious in chest CT images that showed ground-glass shadow combined with interstitial lesions than in patients with ground-glass shadow alone, but the difference between them was not statistically significant (Table 5). The lung function of COVID-19 patients gradually recovered within three months after discharge, which was consistent with the lung imaging findings (Figures 1 and 2). Lung function also improved with the recovery of lung lesions.

## 3. Discussion

Since the outbreak of COVID-19, great attention has been given to the prognosis and outcome of patients worldwide. Pulmonary fibrosis and pulmonary dysfunction were common occurrences of SARS and MERS in the past [7], so whether pulmonary fibrosis and pulmonary dysfunction will develop after patients recover from COVID-19 is also of major concern to the public.

In COVID-19 patients, pulmonary edema, protein exudation, and infiltration of multinucleated giant cells and macrophages in the alveolar lumen were observed in the lung tissue at the early stage [15], followed by bilateral diffuse alveolar injury accompanied by fibromyxoid exudate, formation of hyaline membrane, and infiltration of interstitial inflammatory cells dominated by lymphocytes [16]. A recent study [17] autopsied postmortem lung tissues of COVID-19 patients and found significant pulmonary parenchymal fibrotic remodeling, that is, the characteristics of fibroblast proliferation. The abovementioned pathological changes may be the main reasons for partial lung function damage and imaging abnormalities in COVID-19 patients after being cured and discharged from the hospital. According to the literature [18, 19], there were obvious sequelae in the follow-up of COVID-19 survivors. However, our study showed that the chest imaging absorption of COVID-19 patients improved three months after cure and discharge, and there was no residual pulmonary interstitial fibrosis, such as grid shadow and interlobular septal thickening. Some patients had mild to moderate pulmonary dysfunction, which differed from the existing literature and may be related to the fact that more patients with the common type were included in our study.

Pulmonary function examination in this study indicated that the pulmonary dysfunction of COVID-19 patients mainly manifested as restrictive ventilation dysfunction, small airway dysfunction, and diffuse dysfunction, and the pulmonary dysfunction of severe patients was more common than that of ordinary patients (Table 2), which was consistent with the research results of Mo and James et al. [9, 20] The lung function indicators of the patients declined by varying degrees upon discharge, but the lung function indicators IVC, FEV1, FVC, MEFR 25, MEFR 50, and MEFR 75 gradually improved three months after discharge, indicating that the novel coronavirus had not caused permanent lung damage and the lung damage of the patients improved over time.

Imaging of COVID-19 mainly includes unilateral local or bilateral ground-glass changes, exudation, and consolidation shadows [5, 6]. In this study, the abnormal chest CT features of COVID-19 patients at discharge were mainly patchy shadows (95%) and ground-glass changes (75%), with thickening of interlobular septa, irregular fibrous cord shadows, consolidation shadows, and nodules. The lesions were mostly multilobar involving both lungs, and no significant fibrosis was observed in any patients. This observation is consistent with the report of Pogatchnik et al. [21], who confirmed that the bronchial biopsy pathology of COVID-19 patients showed lung tissue inflammation, which was different from the pathology of pulmonary interstitial fibrosis. According to the case report of Dou et al. [22], two patients with COVID-19 underwent multiple chest CT scans after discharge. The initial CT scans showed multiple patchy ground-glass shadows (GGOs) and a small amount of fibrous tissue in both lungs. During follow-up, the CT scans showed that the density and area of lesions gradually decreased, suggesting that the abnormal lung imaging manifestations gradually improved, which is consistent with the gradual improvement of pulmonary lesions in the 80 patients in this study three months after discharge. Lesions such as ground-glass shadows, grid shadows, and interlobular septal thickening were completely absorbed in 70 patients, leaving only 10 patients (12.5%) with abnormal pulmonary imaging, but the density of abnormal lesions became lighter and the range narrowed.

The correlation analysis between chest CT scans and pulmonary function tests showed that among COVID-19 patients, the degree of small airway function damage in patients with double lung lesions identified by chest imaging was more serious than that in patients with single lung lesions. The pulmonary functions FEV1, FEV1/FVC, IVC, and MEFR 25–75 in patients with ground-glass shadows combined with interstitial lesions decreased more significantly than those with only ground-glass shadows. Therefore, pulmonary function tests and chest CT scans are indispensable examination methods for patients with COVID-19. Especially in patients with severe chest imaging manifestations (bilateral lesions, ground-glass shadow, and interstitial lesions), the damage to pulmonary function is obvious. Therefore, the severity of chest CT scan findings reflects the degree of pulmonary function damage to a certain extent.

After three months of follow-up review after discharge, 87.5% of the patients recovered completely. The remaining lung lesions were also being slowly absorbed at the follow-up review compared with those at discharge. This absorption indicates that pulmonary lesions basically recovered in a short time, and no change in permanent pulmonary fibrosis was observed. This recovery is different from the original SARS survivors [23], which is also different from 23.6% of patients who had pulmonary fibrosis reported in the literature [24]. This difference may be because most of the patients we selected were healthy patients, while the patients included in the research by González et al. [24] were critically ill patients admitted to the ICU. In terms of lung function, most of the patients had varying degrees of lung function impairment when they were discharged from the hospital. After three months, their lung function indicators gradually recovered, and approximately 40% of the patients still had diffusion dysfunction. The degree of lung function damage was consistent with that of chest CT scans. Due to insufficient conditions and data, our study was unable to conduct a comparative analysis of the previous lung function and chest imaging findings of the COVID-19 patients, so the influence of previous lung disease on the current study results cannot be excluded. The DLCO test was not performed at the time of discharge, so the degree of diffusion function damage and recovery could not be analyzed. The limited number of cases (*N* = 80) may limit the generalization of the results. Meanwhile, most of the patients included in our study were healthy, and the subsequent recovery of lung function in severe patients needs to be further studied.

The prognosis and outcome of COVID-19 patients are of great concern. High-resolution thin-slice CT scans can show microscopic lesions in the lungs, which is an ideal examination method for monitoring residual lung lesions. Lung function tests are of great significance in evaluating the prognosis and quality of life of patients. Therefore, it is necessary to conduct high-resolution thin-layer CT scans and lung function follow-up examinations for COVID-19 patients after discharge. The results of this study indicate that the lung imaging lesions and lung function impairment of COVID-19 patients gradually improve with time, and early lung rehabilitation intervention may be a therapeutic strategy, but more studies are needed.

## Figures and Tables

**Table 1:** Characteristics of the patients infected with COVID-19 (*n* = 80).

Characteristic	Results

Age (years)	
Median (IQR)	45.86 ± 11.23
Range	23–79
<30	4 (5%)
30–49	42 (52.5%)
50–69	30 (37.5%)
>70	4 (5%)
Gender	
Men	42 (52.5%)
Women	38 (47.5%)
Smoking history	4 (5%)
Chronic obstructive pulmonary disease	2 (2.5%)
Diabetes history	4 (5%)
History of hypertension	10 (12.5%)
Type	
Severe	24 (30%)
Nonsevere	56 (70%)
Days of symptom occurrence	7.21 ± 2.06
Length of stay	23.93 ± 7.16

Data are *n* (%), *n*/*N* (%), and median.

**Table 2:** Pulmonary function characteristics of COVID-19 patients (*n* = 80).

Pulmonary dysfunction	On the day of discharge			Three months after discharge		
	ALL (*n* = 80)	Severe (*n* = 24)	Nonsevere (*n* = 56)	ALL (*n* = 80)	Severe (*n* = 24)	Nonsevere (*n* = 56)

Ventilation dysfunction	66 (82.5%)	22 (91.7%)	44 (78.6%)	18 (22.5%)	6 (25%)	12 (21.4%)
Small airway dysfunction	48 (60.0%)	16 (66.7%)	32 (57.1%)	28 (35%)	14 (58.3%)	14 (25%)
Ventilation dysfunction with small airway dysfunction	42 (52.5%)	16 (66.7%)	26 (46.4%)	18 (22.5%)	6 (25%)	12 (21.4%)
Disseminated dysfunction				32 (40.0%)	14 (58.3%)	18 (32.1%)

Data are expressed *n* (%) unless specified otherwise.

**Table 3:** Pulmonary function of COVID-19 patients (*n* = 80).

Pulmonary function (%Pred)	On the day of discharge	Three months after discharge	*P* value

FEV1/FVC	99.9 ± 9.6	96.9 ± 17.7	0.377
IVC	62.6 ± 13.8	77.8 ± 16.9	0.006
FVCex	84.3 ± 12.9	92.1 ± 18.9	0.011
FEV1	83.7 ± 12.1	90.4 ± 12.5	0.038
MEFR25	72.3 ± 31.4	78.3 ± 35.5	0.083
MEFR50	81.9 ± 27.3	90.5 ± 24.4	0.047
MEFR75	90.4 ± 19.2	99.1 ± 22.8	0.052
DLCO		92.3 ± 23.0	

Data are expressed as means (standard deviations).

**Table 4:** Radiographic findings of COVID-19 patients (*n* = 80).

Imaging findings	On the day of discharge	Three months after discharge

Normal	2 (2.5%)	70 (87.5%)
Ground-glass opacity	60 (75%)	
Patchy shadowing	76 (95%)	8 (10%)
Interstitial lesions	24 (30%)	
Consolidation shadows	20 (25%)	
Nodular shadows	20 (25%)	2 (2.5%)
Scope of lesions		
On both sides of the lesion	60 (75%)	4 (5%)
Unilateral lesion	20 (25%)	6 (7.5%)

Data are expressed *n* (%) unless specified otherwise.

**Table 5:** Correlation between pulmonary function and pulmonary imaging at discharge.

Pulmonary function (%Pred)	Two lung lesions (*n* = 60)	Single lung lesions (*n* = 20)	*P* value	GGO (*n* = 38)	GGO with interstitial lesions (*n* = 22)	*P* value

FEV1/FVC	97.9 ± 9.3	105.5 ± 8.1	0.027	102.0 ± 10.0	95.0 ± 9.5	0.09
IVC	62.6 ± 13.9	62.9 ± 13.6	0.947	64.2 ± 12.6	56.6 ± 12.4	0.146
FVCex	86.0 ± 12.8	79.5 ± 12.2	0.181	82.9 ± 13.2	85.3 ± 10.9	0.644
FEV1	83.8 ± 12.4	83.4 ± 11.4	0.937	83.9 ± 11.5	80.7 ± 10.8	0.490
MEFR25	69.3 ± 33.8	81.0 ± 31.9	0.045	78.8 ± 39.7	67.6 ± 35.9	0.553
MEFR50	76.6 ± 26.1	97.5 ± 24.9	0.038	78.8 ± 39.7	74.4 ± 28.5	0.438
MEFR75	89.2 ± 20.2	94.2 ± 15.2	0.487	94.5 ± 20.6	84.1 ± 19.6	0.216

## Data Availability

The data used to support this study are available from the corresponding author upon request.
